# Composite t(14;18)-Negative Follicular Lymphoma and Nodular Lymphocyte-Predominant Hodgkin Lymphoma

**DOI:** 10.1155/2018/4312594

**Published:** 2018-08-02

**Authors:** John Patrick O'Neill, Fiona Quinn, Anita Dowling, Jan Walker, Triona Hayes, Brian Bird, Richard Flavin

**Affiliations:** ^1^St. James's Hospital, Dublin, Ireland; ^2^Bons Secours Hospital, Cork, Ireland

## Abstract

A composite lymphoma is the rare simultaneous occurrence of two or more distinct lymphomas within a single tissue or organ. Herein, we describe a case of a 51-year-old man presenting with a history of lower limb rash, fatigue, and bulky abdominopelvic lymphadenopathy. An excisional left iliac lymph node biopsy was notable for the composite presence of two distinct lymphoid neoplasms, nodular lymphocyte-predominant Hodgkin lymphoma (NLPHL), and follicular lymphoma (FL). Multiplex PCR and FISH analyses failed to demonstrate a t(14;18)(q32;q21) translocation in either composite lymphoma component. A clonal light-chain kappa (V/JC intron-kde) gene rearrangement was detected in the FL component only.

## 1. Introduction

Composite lymphomas (CLs) are defined by the rare simultaneous occurrence of two or more distinct lymphomas within one tissue or organ and were first described as an entity in 1954 [1]. It is estimated that CL accounts for approximately 1–4% of all lymphoma diagnoses [2]. CL has been typically found to comprise a classical Hodgkin lymphoma (cHL) and a non-Hodgkin lymphoma (NHL) such as follicular lymphoma (FL) [3], chronic lymphocytic leukaemia (CLL) [4], mantle cell lymphoma (ML) [5], or diffuse large B-cell lymphoma (DLBCL) [6]. Combinations of two NHL subtypes have also been described [7].

A rare occurrence is that of a CL including nodular lymphocyte-predominant Hodgkin lymphoma (NLPHL). Herein, we describe a case of composite FL and NLPHL diagnosed concurrently within the same lymph node.

## 2. Clinical History

A 51-year-old man with a background history of lower limb rash and unexplained fatigue had an incidental finding of bulky abdominopelvic lymphadenopathy during investigation for prostatitis. Skin punch biopsy of the flank and medial left calf noted features of papular urticaria in both specimens. In addition, the left calf biopsy showed features of a mixed septal and lobular panniculitis. Retroperitoneal lymph node core biopsy and open intra-abdominal nodal biopsy at minilaparotomy in another institution failed to yield sufficiently the diagnostic material, and a PET-directed laparoscopic left iliac lymph node excisional biopsy was performed. Following diagnosis, he was treated with bendamustine and rituximab with a complete metabolic response. He has completed 1 of 2-year maintenance rituximab with no clinical evidence of relapse.

## 3. Pathological Findings

**Figure 1:**
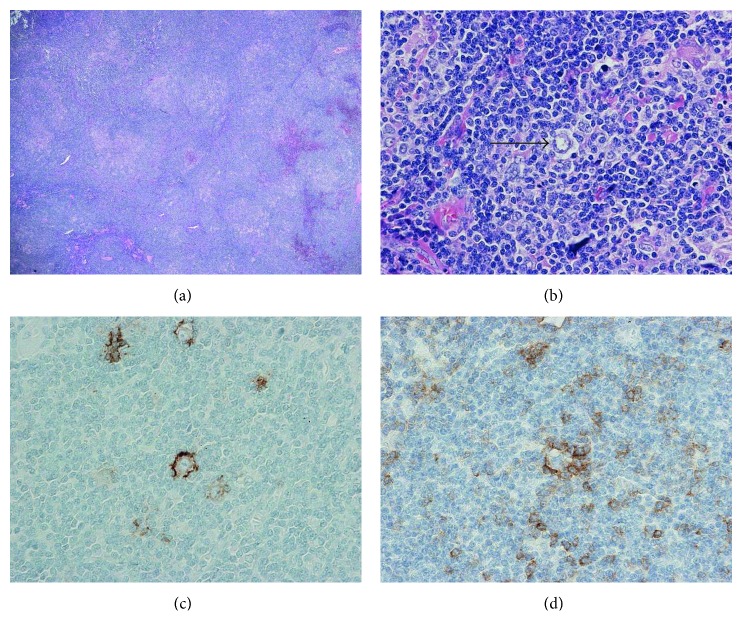
NLPHL. (a) H&E staining, low power, showing vague nodular effacement. (b) H&E staining, high power, showing large LP cells (centre arrow) in a background population of small lymphocytes and histiocytes. (c) Immunostaining for EMA showing membrane positivity in LP cells. (d) Immunostaining for PD-1 showing positive membrane staining of T-cell rosettes surrounding LP cells.

Haematoxylin and eosin (H&E) staining of the lymph node showed one discrete block of the biopsy nodular areas composed of small lymphocytes, epithelioid histiocytes, immunoblasts, and lymphocyte-predominant (LP) cells (Figure 1). There was background progressive transformation of germinal centres (PTGC). The LP cells were positive for immunohistochemical stains CD45, CD20, CD79a, PAX-5, OCT-2, BOB-1, BCL-6, EMA, and MUM-1 and were negative for CD30 and CD15. The background T-cell population was CD4 predominant without aberrant T-cell antigen loss (CD2, CD5, and CD7 positive). PD-1-positive T-cell rosettes were seen around LP cells [8]. The follicular dendritic meshwork was highlighted by CD21 and CD23 staining. EBV-encoded small RNAs in situ hybridization (EBER-ISH) was negative. Overall, the above morphology and immunoprofile were felt to be in keeping with NLPHL. Multiplex polymerase chain reaction (PCR) analysis was performed on a whole discrete section of NLPHL, and a polyclonal immunoglobulin (Ig) gene rearrangement profile was detected. In addition, the t(14;18) BCL2-JH translocation was not detected. Insufficient material was available for FISH analysis and for laser capture microdissection for downstream PCR analysis.

**Figure 2:**
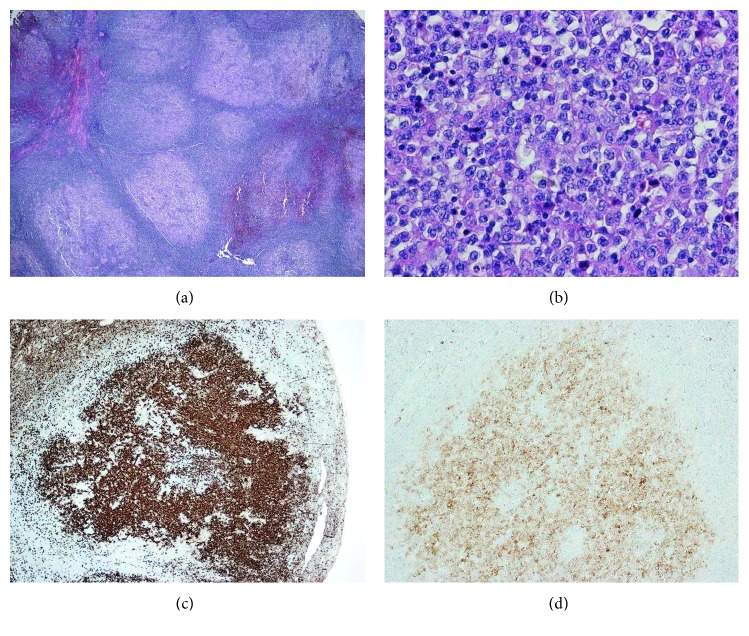
Follicular lymphoma. (a) H&E staining, low power, showing pronounced nodular effacement by closely packed nodules lacking mantle zones. (b) H&E staining, high power, showing neoplastic follicle containing centroblasts (>15 per HPF). Immunostaining for (c) CD20 and (d) CD10 showing follicle positivity.

**Figure 3:**
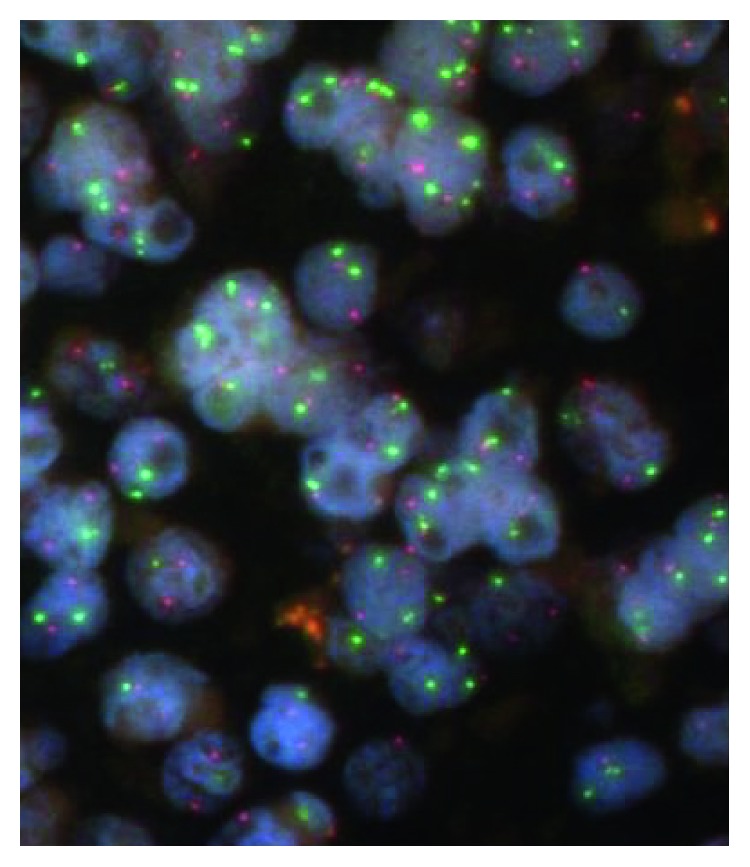
Fluorescence in situ hybridization photomicrograph of the follicular lymphoma component using LSI IGH/BCL2 t(14;18)(q32;q21) dual colour probes showing no evidence of t(14;18) translocation in nonoverlapped nuclei.

In a separate block section of the same lymph node biopsy, there were neoplastic follicles present containing centroblasts (numbering >15 per high-power field) and admixed centrocytes (Figure 2). Immunohistochemistry showed that the neoplastic follicles were positive for CD20, CD79a, CD10, BCL-6, and BCL-2 (weak) with equivocal staining for MUM-1. The Ki-67 proliferation index within follicles was 50%. The morphology and immunoprofile were in keeping with a follicular lymphoma (FL), grade 3a, follicular pattern. Multiplex PCR performed on FFPE tissue from a distinct area of FL detected a clonal Ig light-chain kappa (V/JC intron-kde) gene rearrangement. Multiplex PCR did not detect a t(14;18) BCL2-JH translocation. Fluorescence in situ hybridization (FISH) did not detect either the t(14;18)(q32;q21) translocation or a BCL-2 rearrangement (Figure 3).

## 4. Materials and Methods

The lymph node was fixed in 10% neutral-buffered formalin followed by paraffin embedding. Immunohistochemistry and in situ hybridization were performed routinely following standardized protocols. Antibodies used were CD3, CD4, CD5, CD7, CD8, CD10, CD15, CD20, CD23, CD30, EMA, Ki-67, Pax-5, and Bcl-2 (Ventana); CD21, CD79a, MUM-1, Bcl-6, and PD-1 (Cell Marque); and Oct-2 and Bob-1 (Leica). EBV-encoded small RNAs in situ hybridization (EBER-ISH) was performed on FFPE sections using the fluorescein-conjugated PNA probe (Roche). Fluorescence in situ hybridization analyses for t(14;18) and BCL-2 rearrangements were performed on FFPE samples according to standardized protocols using probe sets LSI IGH/BCL2 t(14;18)(q32;q21) and LSI BCL2 (dual colour) (Abbott Vysis Inc, Downers Grove, IL). t(14;18)(q32;q21) and LSI BCL2 were deemed positive if >10% of nonoverlapped lymphoma nuclei had a demonstrable translocation or break-apart signals, respectively. DNA was extracted from FFPE using Qiagen DNAse kits. Multiplex polymerase chain reaction (PCR) analysis was performed using BioMED primers and probes with PCR products visualized using GeneScan as previously described [9]. Extracted DNA from FFPE was also tested for the presence of the t(14;18) BCL2-JH translocation using multiplex PCR.

## 5. Discussion

This report outlines a second documented case of a composite lymphoma comprising NLPHL and FL. In the previously published case of composite NLPHL and FL, the t(14;18) translocation was detected by FISH, but interestingly, no clonal immunoglobulin gene rearrangements were detected in the FL component, which is in direct contrast to our case.

Cases of composite cHL and FL have been previously described [10–12]. In two of these cases, the cHL and FL components were shown to be clonally related, with shared and distinct Ig V mutations in each instance [10, 11]. This suggests a common origin of the cHL and FL components. Indeed, in an extended series of 19 composite cases involving cHL and other non-Hodgkin lymphomas, where clonality was examined, shared clonality was demonstrated in 12/19 (63%) cases [13]. Furthermore, in four composite cases involving NLPHL, shared clonality has been shown in cases involving DLBCL [14], T-cell-rich large B-cell lymphoma [15], and cHL [16, 17]. The common finding of shared clonality in cases described as composite lymphomas raises the question of whether these lymphomas are in fact “distinct” entities by Custer's 1954 definition [1, 18]. Composite lymphomas where common clonality is present may just represent differing phenotypes of an identical progenitor clone. In our case, we were unable to determine whether shared clonality was present due to insufficient tissue available for laser capture microdissection. In the single other reported instance of composite NLPHL and FL, the authors were also unable to determine the clonal relationship [19].

Finally, composite lymphomas may be more common than previously thought. A 2015 Japanese study examined 154 cHL cases and found a 20% incidence of composite lymphoma in patients ≥40 years [12]. Interestingly, they also noted that marrow involvement by the cHL component and the presence of a high-grade component were adverse prognostic factors.
